# Supplementary material for: An F-ratio-based method for estimating the number of active sources in MEG
Source: Front Hum Neurosci. 2023 Sep 14;17:1235192. doi: 10.3389/fnhum.2023.1235192 (PMC10537939; doi:10.3389/fnhum.2023.1235192)
Supplement: Supplementary file 1 [file Image_1.pdf]

Supplementary Material

An F-Ratio-Based Method for Estimating the Number of Active Sources in MEG

Amita Giri, John C. Mosher, Amir Adler and Dimitrios Pantazis

(a)

Human MEG F-Ratio Value	Optimal F-Ratio Threshold	Null Hypothesis	Decision
$F_{0\to1} = 1.31$	$F^{th}_{0\to1} = 1.01$	$F_{0\to1} > F^{th}_{0\to1}$	'Reject'
$F_{1\to2} = 1.21$	$F^{th}_{1\to2} = 1.05$	$F_{1\to2} > F^{th}_{1\to2}$	'Reject'
$F_{2\to3} = 1.02$	$F^{th}_{2\to3} = 1.03$	$F_{2\to3} < F^{th}_{2\to3}$	'Accept'

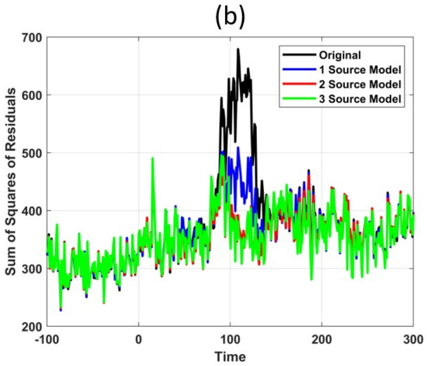

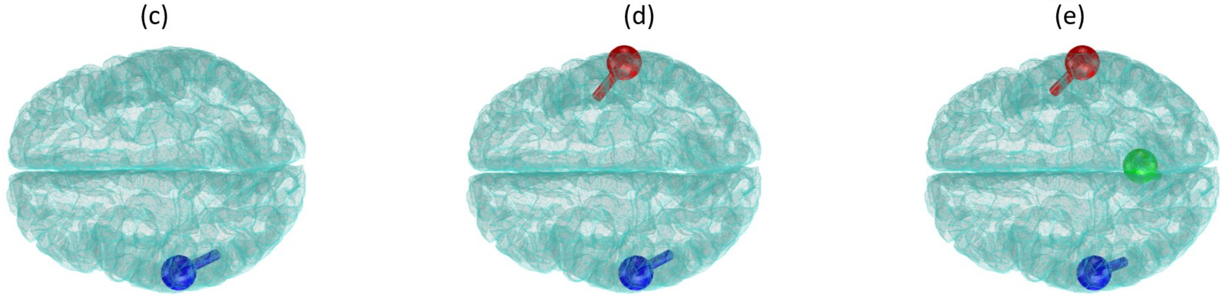

Fig. S1. Performance of the F-ratio method in estimating the number of sources in human auditory data at approximately 0 dB SNR (corresponding to 44 trials). (a) Comparison of obtained F-ratio values in human data with optimal F-ratio thresholds, supporting a model with two active sources. (b) Plot of the sum of squares of residuals for models with different numbers of active sources. (c) Localization of a single source. (d) Localization of two sources. (e) Localization of three sources. Notably, both the Akaike Information Criterion (AIC) and Minimum Description Length (MDL) methods estimated 31 sources. These findings demonstrate the efficacy of the F-ratio method in accurately estimating the number of sources in human auditory data.
